# Clinical Predictors of Abnormal Head Computed Tomography Findings in Non-trauma Patients Presenting to a South African Emergency Department

**DOI:** 10.3389/fradi.2021.759731

**Published:** 2021-10-25

**Authors:** Ekin Simwatachela, John O. Ozoh, Langalibalele H. Mabuza, Chester Kalinda

**Affiliations:** ^1^Department of Diagnostic Radiology and Imaging, Sefako Makgatho Health Sciences University, Pretoria, South Africa; ^2^Department of Family Medicine and Primary Health Care, Sefako Makgatho Health Sciences University, Pretoria, South Africa; ^3^Department of Public Health, College of Health Sciences, University of KwaZulu-Natal, Durban, South Africa; ^4^Bill and Joyce Cummings Institute of Global Health, University of Global Health Equity (UGHE), Kigali, Rwanda

**Keywords:** emergency department (ED), clinical predictors, non-trauma, patients, computed tomography, abnormal head CT findings

## Abstract

**Background:** Head computed tomography (head CT) examinations conducted at emergency departments (EDs) for non-trauma patients are expensive and expose patients to ionizing radiation. Identification of symptoms likely to yield abnormal head CT scans can reduce costs and prevent unnecessary patient irradiation. There is limited comprehensive data in the literature concerning the utilization of head CT in low- and middle-income countries (LMICs) EDs.

**Methods:** A retrospective study of successive non-contrasted head CT scans from February 2017 through January 2018 performed on non-trauma ED patients aged 18 years and above without known pre-existing intracranial pathology was conducted. Univariate and multivariate logistic models were used to determine which presenting clinical features were likely to yield abnormal head CT findings. Clinical information was obtained from the history and physical examination findings entered on the requisition form by the ED clinicians and from previous head CT reports if present on the picture archiving and communication system (PACS).

**Results:** A total of 396 consecutive patients who received head CT examinations had a median age of 49 years (IQR: 36–53), and 53.3% were male (*n* = 211/396). Of the head CT scans included, 73.5% of head CTs included were abnormal (*n* = 291/396). Age >61 years (aOR:1.54; 95%CI: 1.12–2.10), focal neurologic deficit (aOR: 2.46; 95%CI: 1.42–4.26), and loss of consciousness (aOR 2.82; 95%CI: 1.21–6.57) were the predictors of abnormal head CT findings.

**Conclusion:** A head CT scan in a non-trauma patient presenting to an emergency department in a low–middle income country like South Africa is likely to yield abnormal findings if a patient presented with age above 61 years, loss of consciousness, or focal neurological deficit.

## Introduction

The arrival of computed tomography scan has transformed the investigation of diseases affecting the head (1, 2). Easy access, high image quality, non-invasive nature, little time consumed, pressure from patients, and fear of litigation are some of the factors that have led to the rise in the use of head CT in the ED (1–4). However, a majority of the rise in CT utilization in the ED is related to an increase in CT imaging per patient encounter, rather than an increase in total ED patient visits (5).

The increase in the number of head CTs performed in non-trauma patients in EDs has led to multiple public health problems such as the rising costs on the part of the patients and health care systems, increased exposure to ionizing radiation, and incidental findings, some of which may require further imaging or create unnecessary healthcare visits or even procedures. Although diagnostic imaging is vital in clinical decision-making, it continues to be the fastest-growing cost center in most countries (6). For instance, as of 2016, the average cost of a head CT in South Africa ranged between $200 and $400, while it ranged between $400 and $800 in the United States of America (7, 8). Other costs include machine maintenance, psychological health effects of imaging, and cost of personnel and materials (6). The possible carcinogenic effects from ionizing radiation and the rising costs of the increasing number of head CTs in the ED are a concern for policymakers and researchers alike (9).

The studies that have been undertaken in developing countries have shown contrasting outcomes, while data for low- and middle-income countries (LMICs are much more limited. Many studies conducted in countries with high utilization of head CT have indicated that head CTs in non-trauma patients are likely to have abnormal findings if the patients were older than 55 years, had focal neurological deficit, or had a loss of consciousness (6, 10, 11). Furthermore, other authors also suggested that nausea and/or vomiting, history of malignancy, and derangement in coagulation, and comorbidities like hypertension also influenced abnormal head CT scan findings. Bent et al. (12) also showed that headache and dizziness were minor predictors of abnormal head CT findings.

Only 15% of head CT examinations in non-trauma patients have been shown to have an abnormality (10, 11, 13–16). Although several patients continue to be referred for head CT in sub-Saharan Africa and other LMICs, data identifying clinical features that can predict abnormal findings and studies about head CT findings in non-trauma patients presenting to LMIC EDs are limited. Here, we report results identifying clinical features and predictors of abnormal head CT in patients with no history of injury and with no known pre-existing brain pathology presenting to the ED of a tertiary hospital in South Africa, a country representing the LMIC cohort.

## Materials and Methods

### Study Setting

The study took place at the Doctor George Mukhari Academic Hospital (DGMAH), a tertiary hospital in the city of Tshwane (Pretoria). DGMAH is the second-largest hospital in South Africa with a 1,650-bed capacity and has many feeder hospitals, which refer patients to the institution. The hospital services a catchment area with a population of 1.7 million including Bojanala district in North West province and parts of Limpopo province. The hospital also serves as the teaching hospital for the Sefako Makgatho Health Sciences University.

### Study Design and Sampling

This was a retrospective cross-sectional review of all radiological reports of patients who presented to DGMAH hospital emergency department between February 1, 2017 and January 31, 2018. The study included patients who were above the age of 18 years, without a history of trauma, no pre-existing brain pathology, and had a head CT scan performed on them in the ER at DGMAH. To compare this study with previous similar studies, we adopted 18 years and above as the age of inclusion into the study as this was the age used in earlier similar studies conducted by Wang and You (17) and Rampersad and Boodram (18). Records indicating the presence or absence of trauma were obtained from the notes of the clinicians on the request forms while the history of previous brain pathology was obtained from the history taken from the patient on presentation at the ED and previous images on the PACS. Pre-existing brain pathology was defined as a history of stroke, history of brain malignancy, brain metastases, history of intracranial surgery, or any previously documented abnormal head CT findings. Patients with extracranial malignancy without known intracranial metastatic disease were included in the study.

The exclusion criteria included patients whose request forms did not have the age of the patient and presenting symptoms. Furthermore, all files with radiological reports by registrars or any other trainee doctor that were not verified by a consultant radiologist were also excluded.

At the time of the study, the radiology department had two CT scan machines both of which were used to obtain patient head CT images namely, the Philips 128 Slices Ingenuity CT Scanner and the General Electric (GE) 128 Slices Optima CT Scanner. Head CT images from the Philips CT scanner were obtained at 120 KV, 300 mA, 1 mm slice thickness. The GE CT scanner obtained head CT images at 140 KV, 350 mA, and 5 mm slice thickness. The PACS at DGMAH (cares-stream PACS) was used to perform a methodical interrogation of successive patients who had a non-contrast head CT.

**Figure 1:**
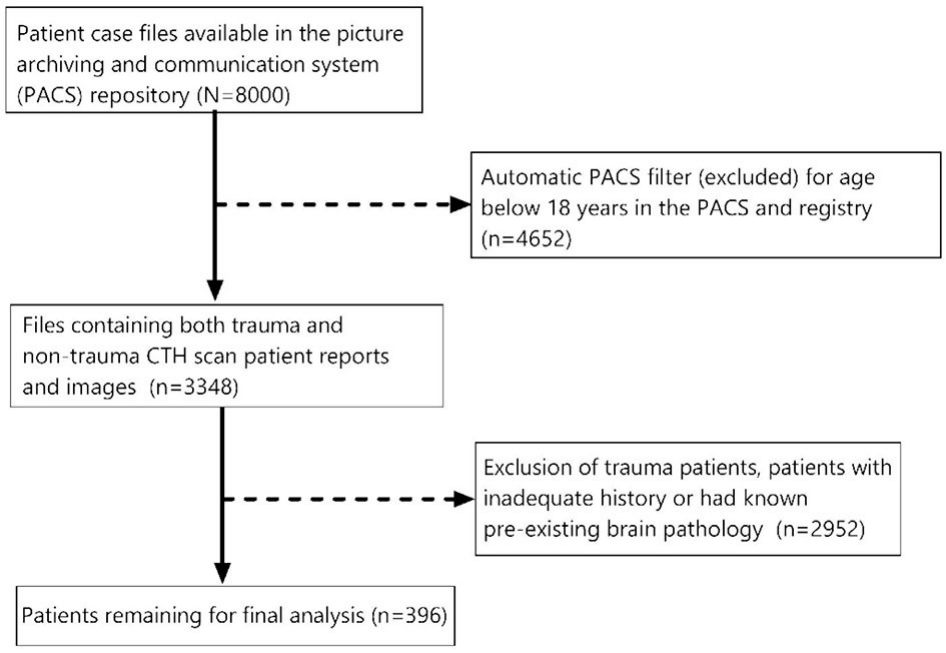
Sampling methods for selection of study participants.

We carried out a census of the number of patient case files available in the PACS repository. Head CT scan reports (8,000) were retrieved from the system for the period under review. Of these, 4,652 were automatically filtered out by the PACS repository because these patients were below 18 years of age. The remaining 3,348 files contained both trauma and non-trauma head CT scan patient reports and images. Of these, 2,952 radiological reports were of trauma patients, did not have patient presenting symptoms, had pre-existing brain pathology, or were head CT reports not verified by a consultant radiologist. This left us with 396 studies to interrogate (Figure 1). All patients in the study had an unenhanced head CT as the initial investigation. However, 10 patients had an enhanced head CT as a follow-up after request by the clinicians.

### Data Collection and Outcome Variable

Data collected were classified into two groups: patient demographic and clinical data. Demographic data collected included the age of the patient and sex. Clinical data were further classified into two groups: Patient symptoms/indication/clinical feature and abnormal head CT findings. The clinical features used as variables in this study were obtained from the radiological reports and included focal neurological deficit (FND) (including paralysis, ataxia, cranial nerve abnormality, dysarthria, tremors, or altered balance), seizure disorder, headache, confusion, nausea/vomiting, loss of consciousness (LOC), neck stiffness, and dizziness. The co-morbidities; diabetes, HIV/AIDS, hypertension, extracranial malignancy, or tuberculosis were also included as independent variables because Rampersad and Boodram (18) showed that comorbidities such as hypertension contribute to abnormal head CT outcomes in ED patients. Not all independent variables used in previous similar studies (12, 13) were included in this study as they could not be gleaned from the information provided by the clinicians in the requisition form. These included but were not limited to; other pathological abnormalities of the posterior fossa, leukocytosis or fever, coagulation profile derangement, general weakness, fatigue, and characterization of seizures into new seizures or known seizure disorder. The primary outcome of interest was an abnormal head CT. An abnormal head CT finding was defined as the presence of an acute infarct, space-occupying lesion, leptomeningeal enhancement, hydrocephalus not caused by age-related cerebral atrophy, dural venous thrombosis, cerebral aneurysm, age-inappropriate cerebral atrophy, complicated paranasal sinus disease (based on Harvard scoring system for rhinosinusitis) (19), pathological intracranial calcifications as defined by Fujioka et al. (20), intracranial hemorrhage, and any other clinically significant finding on the head CT. Only complicated paranasal sinus disease (association with headache or presence of pathological changes such as periorbital cellulitis or intracranial collections) was considered as an abnormal finding (21).

For this study, cerebral, cerebellar, and basal ganglia calcifications were considered abnormal in symptomatic patients in a setting of suspected infection like neurocysticercosis, metabolic diseases like hypoparathyroidism, vascular malformation, and neurodegenerative disease in keeping with the study by Fujioka et al. (20). Nonconcerning calcifications in the pineal gland, habenula, choroid plexus, dura, falx, tentorium cerebelli, petroclinoid ligaments, superior sagittal sinus, and dentate nuclei were considered normal (20). Based on the features observed (normal/abnormal), we decided to convert the outcome into a dichotomous variable.

### Data Analysis

Descriptive analyses were performed using Stata statistics software (Release 14. College Station, TX: StataCorp LP). Associations between socio-demographic and clinical features which were the predictor variables and primary outcomes (normal/abnormal head CT outcome) were statistically assessed using bivariate analysis. Furthermore, we fit a univariate logistic regression with clinical and demographic variables (age and sex) as covariates to determine the significance of the relationships observed in the bivariate analysis. Variables that had a *p* < 0.15 in bivariate analysis were included in the univariate model, while only those that were statistically significant in the univariate model were included in the multivariate logistic model (22, 23). For our final multivariate model, we fit a full model with all significant variables from the univariate models. Then, using a backward selection method, we excluded each univariably assessed covariate based on its significance level. Covariate stepwise selection continued until the model become adequate. We assessed the goodness of fit of the final model using the Hosmer–Lemeshow test (24).

## Results

A total of 396 (*n* = 396) head CT reports were evaluated. The median age of the patients was 49 years [interquartile range (IQR): 36–53 years]. Study participants were categorized into three age groups (18–40, 41–60, and 61–100 years). Of the three age groups, those aged between 41 and 60 years comprised 37.6 % (*n* = 149), while the least (*n* = 117, 29.5%) were aged between 61 and 100 years. The majority (*n* = 211, 53.3%) of the patients were males (Table 1). In a bivariate analysis, age, FND, seizure, LOC, and hypertension were significantly associated (*p* < 0.05) with the abnormal head CT findings. On the other hand, sex, headache, renal failure, altered mental status, HIV/AIDS infection, neck stiffness, diabetes, and extracranial malignancies were not associated with abnormal head CT findings (Table 1).

**Table 1:** Association between the outcome variables and demographic and clinical features.

**Variable**		**Normal CT finding**	**Abnormal CTH findings**	***p*-value**
Age	18–40 years	48 (36.92%)	82 (63.08%)	**0.001**
	41–60 years	40 (26.85%)	109 (73.15%)	
	61–100 years	17 (14.53%)	100 (85.47%)	
Sex	Female	45 (24.32%)	140 (75.67 %)	0.335
	Male	60 (28.44%)	151 (71.56%)	
Focal neurological deficit (FND)	Absent	83 (33.07%)	168 (66.93%)	**0.001**
	Present	22 (15.17%)	123 (84.83%)	
Seizure	Absent	82 (24.62%)	251 (75.38%)	**0.05**
	Present	23 (36.51%)	40 (63.49%)	
Headache	Absent	85 (26.40%)	237 (73.60%)	0.912
	Present	20 (27.03%)	54 (72.97%)	
Renal failure	Absent	103 (26.41%)	287 (73.59%)	0.703
	Present	2 (33.33%)	4 (66.67%)	
Confusion	Absent	64 (24.90%)	193 (75.1%)	0.323
	Present	41 (29.5%)	98 (70.5%)	
Nausea and/or vomiting	Absent	100 (27.17%)	268 (72.83%)	0.282
	Present	5 (17.86%)	23 (82.14%)	
Loss of Consciousness (LOC)	Absent	98 (28.65%)	244 (71.35%)	**0.015**
	Present	7 (12.96%)	47 (87.04%)	
Neck stiffness	Absent	95 (25.61%)	276 (74.39%)	*0.115*
	Present	10 (40%)	15 (60%)	
Dizziness	Absent	102 (26.15%)	288 (73.85%)	0.189
	Present	3 (50%)	3 (50%)	
Diabetes	Absent	100 (27.1%)	269 (72.9%)	0.329
	Present	5 (18.52%)	22 (81.48%)	
HIV/AIDS	Absent	96 (27.51%)	253 (72.49%)	0.223
	Present	9 (19.15%)	38 (80.85%)	
Hypertension	Absent	98 (29.08%)	239 (70.92%)	**0.006**
	Present	7 (11.86%)	52 (88.14%)	
Malignancy	Absent	103 (26.41%)	287 (73.59%)	0.703
	Present	2 (33.33%)	4 (66.67%)	

*The bold values are those that were associated (p < 0.05) with the abnormal head CT findings in the bivariate analysis, (age, FND, seizure, LOC, and hypertension)*.

Results of the univariate analysis are shown in Table 2. Age (OR: 1.71; 95% CI: 1.29–2.27), focal neurological deficit (FND) (OR: 2.76; 95% CI: 1.63–4.67), loss of consciousness (LOC) (OR: 2.69; 95% CI: 1.17–6.17), and hypertension (OR: 3.04; 95% CI: 1.33–6.93) were observed to be statistically significantly associated with abnormal head CT findings. It was further observed that patients in the age group of 61–100 years were 3.44 (95% CI: 1.84–6.43)-fold likely to have an abnormal head CT finding compared with those aged between 18 and 40 years. We also observed that patients with FND (OR: 2.76; 95% CI: 1.63–4.67), LOC (OR: 2.70; 95% CI: 1.17–6.17), and hypertension (OR: 3.04; 95% CI: 1.33–6.94) were more likely to have abnormal head CT findings than those without these conditions. We also observed that seizure (OR: 0.57; 95% CI: 0.32–1.00) and neck stiffness (OR: 0.52; 95% CI: 0.22–1.18) were not significantly associated with head CT findings (Table 2).

**Table 2:** Factors associated with normal/abnormal head computed tomography (CT) findings used in univariate and multivariate analysis.

	**Univariate analysis**			**Multivariate analysis**		
**Variable**	**OR (Unadjusted)**	***P*-value**	**96% CI**	**OR(Adjusted)**	***P*-value**	**96% CI**
18-40 years	Reference					
41-60 years	1.60	0.072	0.96-2.65	1.27	0.38	0.75-2.16
61-100 years	**3.44**	**0.001**	**1.84–6.43**	**2.51**	**0.01**	**1.31–4.82**
Without FND	Reference					
With FND	**2.76**	**0.001**	**1.63–4.67**	**2.46**	**0.00**	**1.42–4.26**
Without seizure	Reference					
With seizure	0.57	0.052	0.32–1.00			
Without LOC	Reference					
With LOC	**2.70**	**0.019**	**1.18–6.17**	**2.92**	**0.01**	**1.25–6.83**
Without neck stiffness	Reference					
With neck stiffness	0.52	0.12	0.22–1.19			
Without hypertension	Reference					
With hypertension	**3.05**	**0.008**	**1.34–6.94**			

*The bold values are the p < 0.05, CI and OR for the variables that were significantly associated with the abnormal head CT findings in the univariate and multivariate analysis*.

In the final multivariable analysis (Table 2), age (aOR: 1.54; 95% CI: 1.12–2.10), FND (aOR: 2.46; 95% CI: 1.42–4.26), and LOC (aOR: 2.82; 95% CI: 1.42–6.57) were significant predictors of head CT findings. We further observed that patients aged 61–100 years were 2.51 (95% CI: 1.31–4.83) likely to have abnormal head CT findings compared with those aged 18–40 years. We also observed that those who had experienced a LOC and those with FND were 2.92 (95%CI: 1.25–6.83) and 2.46 (95% CI: 1.42–4.27) times, respectively, likely to have abnormal head CT findings (Table 2). The Hosmer and Lemeshow goodness-of-fit chi-squared test of the model was 0.54 (*p* = 0.99).

We observed 10 abnormal findings (Table 3). The abnormal head CT findings in patients were space-occupying lesion, leptomeningeal enhancement, pathological intracranial calcifications, complicated paranasal sinus pathology, hydrocephalus, dural venous thrombosis, cerebral aneurysm, and age-inappropriate cerebral atrophy. The least common abnormal head CT findings were dural venous sinus thrombosis (*n* = 2, 0.69%), cerebral aneurysm (*n* = 4, 1.37%), and leptomeningeal enhancement (*n* = 6, 2.06%). Paranasal sinus pathology (*n* = 78; 26.8%) and acute infarct (*n* = 76; 26.12%) were the main abnormal findings observed. Ten patients (*n* = 10/396 or 2.5 %) had a follow-up enhanced head CT upon request by the clinician and the findings included cerebral artery aneurysm, leptomeningeal enhancement, and dural venous thrombosis. The other case of dural venous thrombosis was diagnosed on non-contrast head CT. Furthermore, the study observed that 90 patients had more than one abnormal findings. Of these, 65 had two abnormal findings, 22 had three abnormal findings, and three had four abnormal findings. The study focused on determining abnormal and normal head CT exams. The analytical aspect did not consider the effect of the multiplicity of abnormal findings.

**Table 3:** Abnormal findings observed in patients with normal and abnormal head CT.

**Variable**	**Abnormal CTH scan**	
Space occupying lesion	Absent	263 (90.38%)
	Present	28 (9.62%)
Leptomeningeal enhancement	Absent	285 (97.94%)
	Present	6 (2.06%)
Pathological calcification	Absent	255 (87.63%)
	Present	36 (12.37%)
Complicated paranasal sinus disease	Absent	213 (73.2%)
	Present	78 (26.8%)
Hydrocephalus	Absent	270 (92.78%)
	Present	21 (7.22%)
Dural venous thrombosis	Absent	289 (99.31%)
	Present	2 (0.69%)
Aneurysm	Absent	287 (98.63%)
	Present	4 (1.37%)
Intracranial hemorrhage	Absent	259 (89%)
	Present	32 (11%)
Acute infarct	Absent	215 (73.88%)
	Present	76 (26.12%)
Age inappropriate cerebral atrophy	Absent	251 (86.25%)
	Present	40 (13.75%)

*Please note that most of the abnormal head CT studies had more than one abnormality and each abnormal finding was expressed as a proportion of the total number of abnormal studies (291)*.

The ratio of abnormal and normal studies was compared with the age interval of the patients and the descriptive statistics are as shown below (Table 4). The presence of the three predictors, namely, age above 61 years, LOC, and FND had a 93% sensitivity in predicting abnormal head CT findings. Our sensitivity study showed that there would be a reduction of 31.8% in the number of head CTs performed if only patients with the predictors were scanned. This shows the possible cost reduction benefits of implementing a prediction protocol.

**Table 4:** Sensitivity of variables for prediction of abnormal head CT.

**Parameter**	**Sensitivity**	**Patients who would not be scanned**
At least one of two clinical predictors^*^	170 of 291 (58.4%)	226 of 396 (57.1%)
At least one of two clinical predictors or age >61	270 of 291 (93%)	126 of 396 (31.8%)

* *Focal neurological deficit and loss of consciousness.*

*The first row in the last column shows the number of patients who did not have the two predictors (excluding age). Two hundred and twenty six (57.1%) patient radiological reports did not have the two predictors and this corresponds (hypothetically speaking) to the number of patients who would not have been scanned if the predictor model was used to decide which of the 396 patients would be scanned. The second row in the last column shows the number of patients who did not have the three clinical predictors and this corresponded to 126 (31.8%) patients not being scanned if the model was used to decide who would be scanned*.

## Discussion

In this exploratory study of ED patients without a history of trauma who underwent head CT, our results showed that patients aged above 61 years, patients with FND, and patients with LOC were likely to have abnormal head CT findings. Although age was observed to be a factor, it carries with it several comorbidities increasing its likelihood of being associated with the abnormal head CT. Furthermore, the results observed in the current study show that the sensitivity of the predictors if one of two clinical predictors or age >61 were applied in a model would be 93%. On the other hand, previous studies by Rampersad and Boodram (18) and Wang and You (17), which had more than five predictor variables in their final model recorded a sensitivity of 94% to 98% in their derivative cohort. Our results further indicate that if variables observed to influence abnormal head CT were implemented on the patients included in the study, 126 patients would not be scanned because they did not have the predictors of abnormal head CT determined in this study. Based on the approximate cost of a head CT of US$400 in South Africa at the time of the study, this would translate into a saving of approximately US$50,000 for the local health care system. Furthermore, these patients would have also avoided the ionizing radiation exposure and apprehension that comes with a head CT.

In our study, we observed that the routine use of head CT for investigation of patients based on the lone presentation of clinical symptoms such as confusion, seizures, neck stiffness, headache, dizziness, and nausea or vomiting had a low likelihood of yielding abnormal head CT findings if FND and LOC were absent and age of the patient was less than 61 years. These findings corroborate the observations made by other investigators who suggested that head CT in patients who were not presenting with symptoms that were predictors of abnormal head CT findings are expensive and had a low yield of abnormal head CTs (6, 10, 12, 25).

Wasay et al. (6) observed that head CT performed on 200 patients presenting with acute dizziness or vertigo did not yield any abnormal head CT findings despite spending an estimated $60,000. Furthermore, Grossman et al. (10), while investigating the utilization of head CT for patients presenting with syncope, found that only five patients (4.4 %) of 113 who had a head CT had abnormal findings. Further analysis of these patients indicated that two patients had abnormal neurological examination, acute headache was noted in one patient, and two other patients had a history of trauma. These authors also suggested a possibility of reducing the performance of head CT in patients with a history of syncope by 56%.

Our results suggest that head CT based on a lone clinical presentation of non-predicting clinical features may have low yields of abnormal head CT, increases the costs on patients and the health care system, and exposes the patients to unnecessary ionizing radiation increasing the risks of cancer development. Therefore, the development of a head CT protocol for non-trauma patients presenting to the ED may be vital in helping reduce associated costs and ionizing radiation exposure.

In keeping with our study, several studies have indicated that most non-trauma patients with abnormal head CT findings usually have a neurological deficit (16, 26). For instance, Naughton et al. (11) showed that of the 15% of patients that had abnormal head CT in their study, 95% had neurological deficits on examination. These authors further observed that two of the patients who did not have positive neurological findings had a history of falling without evidence of delirium, and this has been observed in several other studies (13, 16, 26, 27).

Although headache is a common presenting clinical feature that may make clinicians order a head CT, we observed that it was not a predictor of abnormal head CT findings. There are contradictory recommendations by emergency medicine physician groups regarding head CT for patients with headaches (27). Emergency physician consensus groups in the United States of America strongly advise urgent non-contrast CT head for patients presenting with headache and new neurological deficits on examination (27). Because certain features of headache are predictive of subarachnoid hemorrhage (SAH) such as the time to peak (28), the emergency physician consensus groups also advise urgent head CT in patients presenting with acute severe headache (27). Investigation of SAH can be challenging because some patients with SAH may only present with headaches and be neurologically intact. Emerging evidence by Perry et al. (29) shows that the Ottawa SAH Rule has 100% sensitivity, whereas neuroimaging has a sensitivity of about 87% in detecting SAH (29). In our study, all patients with intracranial hemorrhage and space-occupying lesions had at least one of the predictors as an indication for the head CT.

We observed that confusion was not a predictor of abnormal head CT findings. This is in agreement with an earlier study by Bennimahadeo and Maharajh (7), which was carried out at a South African Institution. We further observed that seizures, as a lone presenting complaint, are not predictors of head CT abnormal findings. There is conflicting information in the literature concerning eligibility for head CT for patients presenting to the ED with seizures. According to Jagoda and Gupta (30), patients presenting for the first time with seizures should not undergo a head CT unless they have co-morbidities such as FND or abnormal baseline mental status. The current evidence shows that patients presenting with seizures usually have other presenting features such as FND or history of malignancy (30, 31).

Our results showed that HIV infection was not a predictor of abnormal head CT. This is in keeping with the study by Ozoh et al. (31) who studied head CT findings in HIV/AIDS patients presenting to DGMAH. These authors attributed the low prevalence of abnormal head CT findings in HIV patients to highly active antiretroviral therapy (HAART) increasing CD4 counts in the population. Furthermore, HIV patients with CD4 counts of 200 cell/mm^3^ or more may not benefit from a head CT but would benefit from MRI brain.

### Strengths and Limitations

While this study has presented independent predictors, other studies in Africa have interrogated individual clinical features rather than the whole spectrum. The other strength of the study was the moderately large pool from where studies qualifying for interrogation were selected. The study had limitations, which included the fact that patients who did not have a head CT were not included in the study. This meant that the actual outcome of administering the clinical predictors could not be evaluated. The other limitation was the lack of comprehensive information on the clinician requisition forms. For instance, it was difficult to understand what vague terms like “drowsiness,” “altered level of consciousness,” or “not able to communicate” meant, so we grouped these symptoms into “loss in consciousness” (to include syncope, unarousable patients, and loss of consciousness). Delirium, confusion, or psychosis were grouped under confusion. The retrospective nature of the study meant that we could not correct for patients left out of the study due to lack of clearly indicated presenting symptoms and lack of history. This also meant that we could not ascertain how acute the headache was in the patient cohort or if the patient had new seizures or had a known seizure disorder as the clinicians generally reported the presenting symptom but did not indicate detailed features of the symptom on the head CT request form. When the automatic PACS filter was used to select head CT reports based on age, 4,652 (58%) studies were filtered out by the system. This could have been a true reflection of the records or could have been due to technical or data entry issues beyond the control of the researchers. It could not be ascertained if this was due to missing information such as date of birth not being entered by the data entry clerk.

Based on the exclusion criteria our study sample of 396 was only 5% of the initial 8,000 study population. If there was any selection bias in the excluded studies, it could have affected the results of the study.

## Conclusion

This study contributed to the literature on head CT utilization in LMICs by showing that the predictors of abnormal head CT findings at a LMIC ED were age above 61 years, FND and LOC. Based on our predictors, 31.8% of head CT would be avoided if head CT was done only in patients who had these predictors, and this would translate into the DGMAH health system saving approximately US$50,000. The nearly 32% of reduction in cases will decompress the ED volumes and reduce patient apprehension in several cases. If this work is validated in a prospective study, it can contribute to the construction of a protocol for patients requiring a head CT and help to save the limited resources and avoid unnecessary ionizing radiation exposure.

## Data Availability Statement

The raw data supporting the conclusions of this article will be made available by the authors, without undue reservation.

## Author Contributions

ES designed the study, collected the data, and wrote the initial manuscript. LM and JO contributed to designing the study and supervision of the work. CK analyzed the data, assisted in writing the initial manuscript, and contributed to the overall arrangement of the article. All authors approved the submission of the final manuscript.

## Author Disclaimer

The views expressed in the submitted article are our own and not an official position of the Sefako Makgatho Health Sciences University and DGMAH.

## Conflict of Interest

The authors declare that the research was conducted in the absence of any commercial or financial relationships that could be construed as a potential conflict of interest.

## Publisher's Note

All claims expressed in this article are solely those of the authors and do not necessarily represent those of their affiliated organizations, or those of the publisher, the editors and the reviewers. Any product that may be evaluated in this article, or claim that may be made by its manufacturer, is not guaranteed or endorsed by the publisher.
